# Cross-cultural adaptation, reliability and validity of the Spanish version of the long-term quality of life questionnaire

**DOI:** 10.3389/fonc.2024.1375125

**Published:** 2024-03-19

**Authors:** Beatriz León-Salas, Amaia Bilbao-González, Ana María de Pascual y Medina, Magdalena Esteva, Ana Toledo-Chávarri, Claudio Fuentes-Sánchez, Uriel Bohn-Sarmiento, Pilar Padrón-Peña, Sonia González-Sánchez, Rafael Valcárcel-López, María del Mar Trujillo-Martín

**Affiliations:** ^1^ Canary Islands Health Research Institute Foundation (FIISC), Santa Cruz de Tenerife, Spain; ^2^ Research Network on Chronic Diseases, Primary Care, and Health Promotion (RICAPPS), Carlos III Health Institute (Instituto de Salud Carlos III), Madrid, Spain; ^3^ Osakidetza Basque Health Service, Basurto University Hospital, Research and Innovation Unit, Bilbao, Spain; ^4^ Research Unit, Kronikgune Health Services Research Institute, Barakaldo, Spain; ^5^ Department of Medicine, Faculty of Health Sciences, University of Deusto, Bilbao, Spain; ^6^ Evaluation Unit (SESCS), Canary Islands Health Service (SCS), Santa Cruz de Tenerife, Spain; ^7^ Majorca Primary Care Management. Research Unit, Palma de Mallorca, Spain; ^8^ Health Research Institute of Balearic Islands (IdIsBA), Palma de Mallorca, Spain; ^9^ Department of Medical Oncology, Nuestra Señora de Candelaria University Hospital, Santa Cruz de Tenerife, Spain; ^10^ Department of Medical Oncology, Gran Canaria Dr. Negrin General University Hospital, Las Palmas de Gran Canaria, Spain; ^11^ Nursing Service, University Hospital of Canary Islands, Santa Cruz de Tenerife, Spain; ^12^ Canary Islands Primary Care, Canary Islands Health Service (SCS), Santa Cruz de Tenerife, Spain

**Keywords:** breast cancer survivors, LTQL, psychometric, rasch measurement, quality of life, Spain

## Abstract

**Purpose:**

The aim of this study was to translate, culturally adapt, and evaluate the psychometric properties of the Spanish Long-Term Quality of Life (LTQL) questionnaire.

**Methods:**

The LTQL was initially translated into Spanish and cross-culturally adapted based on established guidelines. The Spanish LTQL was administered to patients with breast cancer who had completed their initial treatment 5 years earlier, along with other self-report measures: Quality of Life in Adult Cancer Survivors (QLACS), Hospital Anxiety and Depression Scale (HADS) and EORT-QLQ-BR23. Reliability was evaluated using internal consistency and test-retest. Convergent and known-groups validity were examined. Structural validity as determined by confirmatory factor analysis (CFA) and Rasch analyses was used to assess the unidimensionality and item-functioning of the LTQL domains.

**Results:**

Cronbach’s alpha were above 0.7 in all domains. Test-retest coefficients were between 0.72 to 0.96 for LTQL domains. LTQL total score was correlated with others total scores of other measures: QLACS (r=-0.39), HADS depression (r=-0.57), HADS anxiety (-0.45) and EORTC-QLQ-BR23 (r=-0.50). CFA provided satisfactory fit indices, with RMSEA value of 0.077 and TLI and CFI values of 0.901 and 0.909, respectively. All factor loadings were higher than 0.40 and statistically significant (P<0.001). Rasch analysis showed that Somatic Concerns domain had 4 misfitting items, and Philosophical/Spiritual View of Life and social Support domains only 1 misfit item. However, unidimensionality was supported for the four domains.

**Conclusion:**

The findings support the validity and reliability of the Spanish version of LTQL questionnaire to be used in long-term cancer female survivors.

## Introduction

Breast cancer (BC) is not only the most common cancer but also one of the most significant health concerns for women due to its high prevalence, morbidity, mortality and impact on healthcare services. In fact, it stands as the leading cause of death among women in Europe (1). According to the European Cancer Information System (ECIS), a total of 355,457 new BC cases were diagnosed in Europe in 2020, of which 34,088 occurred in Spain (2).

The number of BC survivors in Europe has been steadily increasing, thanks to advancements in diagnostic techniques, and early detection and treatment (3). A long-term cancer survivor is typically defined as the individual who surpasses the five-year mark after cancer diagnosis (4), as studies have shown that cancer relapses are more frequent within the initial five years post-diagnosis (5).

Approximately 1,900,000 individuals in South Europe have survived cancer, accounting for 1.5% of the population. Among all types of cancer, BC exhibits the highest survival rates (6). As of 2020, it was estimated that there were 144,233 cases of BC survivors at the five-year mark (7). While the overall five-year cancer survival rate in Spain exceeds 50% for adult patients (3). The five-year survival rate for BC was estimated at 85.2% from 2010-2014 (8).

Given the high survival rates among breast cancer patients, it is crucial to conduct comprehensive assessments of their long-term quality of life (QoL) in order to effectively address their needs. QoL is defined as the subjective well-being associated with happiness and personal satisfaction with life as a whole (9). This definition encompasses various domains, including physical, psychosocial, and spiritual aspects, which Ferrell et al. identified in 1995 as the most significant aspects when evaluating the QoL of BC (10). Currently available specific QoL instruments mainly focus on the physical and psychosocial domains, which are more relevant during the early stages of the disease, while neglecting the spiritual domain. However, women with BC often emphasize the importance of spirituality in their experience with the disease (11, 12). Spirituality involves individuals experiencing a connection with themselves, others, nature, and/or a higher power or sense (13). Recent reviews have indicated that spirituality can aid patients in coping with their illness experience while reducing depression and anxiety (14).

In recent decades, several specific QoL questionnaires for patients with cancer have emerged as important assessment tools (15). Among these, the Long-Term Quality of Life questionnaire (LTQL) (16) was developed by Wyatt and Friedman for women who experience long-term cancer.

Wyatt and Friedman discovered that women who were five years post-diagnosis experienced minimal physical changes, yet they encountered significant impacts on the psychological, social, and spiritual aspects (16). As a result, they developed the LTQL, an instrument specifically for this population (17). The LTQL comprises four domains of QoL: somatic concerns, physical fitness, social support, and philosophical/spiritual vision of life. Importantly, the LTQL encompasses a broad and existential conceptualization of spirituality that extends beyond religious preferences, making it suitable for assessing this domain. The instrument was validated by the authors using a sample of 188 long-term cancer survivors from the United States (17).

The objectives of the present study were: 1) to perform a cross-cultural adaptation of the LTQL questionnaire for use in Spain and 2) to conduct a validation study of the LTQL, analyzing the internal structure using classical test theory such as exploratory factor analysis (EFA) and confirmatory factor analysis (CFA), as well as Rasch analysis; to assess known-groups validity, convergent validity, and reliability of the different domains of the LTQL.

## Methods

This was a cross-sectional study conducted between 2019 and 2021. The study protocol was approved by the institutional review board of the Nuestra Señora de Candelaria University Hospital (approval number CHUNSC-2019-04).

### Study population and data collection

Participants were recruited from patient associations and primary care centers of Canary Islands, Spain. All participants were provided with information about the study and were asked to participate voluntarily. All the eligible subjects were female adults with BC who had completed their initial cancer treatment five years prior to the start of the study and provided informed consent to participate in the study.

They were given the option to complete the questionnaires in person or via mail.

A sample size was calculated based on recommendation of 10 participants for each questionnaire item for which validity is desired. In this case, 10 multiplied by 33 equals 330. Finally, a sample of 340 participants was obtained.

### Measures

Participants were asked to complete the Spanish version of the LTQL and other Spanish validated questionnaires, namely the Quality of Life in Adults Cancer Survivors (QLACS) (18), European Organization for Research and Treatment of Cancer Quality of Life questionnaire module for Breast Cancer Patients (EORTC-QLQ-BR23) (19) and the Hospital Anxiety and Depression Scale (HADS) (20). Sociodemographic and clinical data were also collected, including gender, age, marital status, education, employment status, years of survival and type of BC treatment.

The LTQL questionnaire (16) consists of 34 items that assess four domains: somatic concerns (14 items), philosophical/spiritual view of life (11 items), fitness (5 items) and social support (4 items). Each item is rated on a five-point Likert scale, ranging from 0 (not at all) to 4 (very much). Some of the items are reverse-scored (items 1, 7, 8, 11, 12, 14, 19, 22, 23, 24, 25, 28, 32, and 34). The range of scores for each domain range from 0 to 4. A high score (i.e. 4) is indicative of high QoL.

The QLACS questionnaire (21) consists of 47 items that assess 12 domains. There are 7 generic domains, each containing 4 items: negative feelings, positive feelings, cognitive problems, sexual problems, pain, fatigue, and social avoidance. Additionally, there are 5 cancer-specific domains: appearance concerns (4 items), financial problems (4 items), distress about recurrence (4 items), family-related distress (3 items) and benefits of cancer (4 items). Each item is rated on a seven-point frequency scale, ranging from 1 (never) to 7 (always), reflecting the frequency experienced in the past four weeks. The domain scores range from 4 to 28, with higher scores indicating lower health-related quality of life (HRQoL). The score for the family-related distress domain is multiplied by 1.33 to compensate the fact that this domain consists of fewer items compared to other domains. The score for positive feelings is reversed as well as item 1 in the fatigue domain. The total score ranges from 28 to 196, and the cancer-specific summary score ranges from 16 to 112. The QLACS has been translated into and validated in Spanish (18).

The QLQ-BR23 (19) is the breast module of the EORTC questionnaire. It consists of 23 items that are divided into four functional scales and four symptom scales. Each scale and single-item measure ranges from 0 to 100. For the functional scales, a high score indicates a high or healthy level of functioning. On the other hand, for the symptom scales, a high score indicates a high level of symptomatology or problems. QLQ-BR23 questionnaire has been translated into and validated in Spanish (22), ensuring its applicability and accuracy in the Spanish-speaking population.

The HADS (23) is a questionnaire that consists of two subscales: anxiety and depression. Each subscale contains 7 questions related to symptoms of anxiety or depression, respectively. Each of the 14 items in the questionnaire is rated on a 4-point Likert scale, ranging from 0 to 3, indicating the severity of symptoms experienced in the previous week. The scoring for each subscale ranges from 0 to 21, with higher scores indicating a higher level of anxiety of depression. The HADS questionnaire has been translated into Spanish and validated (24).

### Development of the Spanish version of the LTQL

We obtained permission from the original authors of the LTQL questionnaire (16) to translate and validate it for use in our study. Following established guidelines for cross-cultural adaptation (25), we carried out the translation and cultural adaptation process.

Two professional translators, whose native language was Spanish, independently translated the original English questionnaire into Spanish. It should be noted that the translators encountered challenges in finding Spanish expressions that were conceptually equivalent to the original expressions. The two translations were then compared and discussed in a meeting that involved the first author of study and the translators. Though this collaborative process, a consensus was reached, resulting in a single adapted version of the questionnaire (version 1.0).

To assess the equivalence of Spanish version 1.0 with the original questionnaire, it was independently back-translated to English by two native professional translators who were highly fluent in Spanish. The back-translations were then compared with the original English version, and any necessary modifications were made to ensure the accuracy and consistency of the Spanish LTQL. To evaluate the understanding of the items by the target population and to assess content validity, the Spanish LTQL was tested on a sample of 7 long-term breast cancer patients. By following this rigorous translation and adaptation process, we aimed to ensure the linguistic and conceptual equivalence of the Spanish version of the LTQL for use in our study.

### Statistical analysis

The unit of analysis was the patient. Descriptive statistical analysis was performed using frequency tables to summarize categorical variables, while means and standard deviations (SDs) were used to summarize continuous variables. For the Spanish version of the LTQL, the distribution of scores was assessed though various measures. These included calculating the mean and SD to determine the average score and its variability across the sample. Additionally, the proportion of patients with one or more missing items was examined to identify any potential issues with item completion. The observed range of scores was also analyzed to understand the spread of scores within the sample. To assess the ceiling an floor effects, which indicate the extent to which scores cluster at the highest and lowest possible values, we compared the distribution of scores with the accepted threshold of <15% (26).

### Reliability

#### Internal consistency

To assess the internal consistency of the questionnaire, we calculated Cronbach’s alpha coefficient (27). This coefficient measures the extent to which the items within each scale of the questionnaire are interrelated. A Cronbach’s alpha coefficient > 0.7 is generally considered acceptable, suggesting good internal consistency (28). We also estimated the McDonald’s omega coefficient (ω), for which a value > 0.70 was considered acceptable (29, 30).

Further, the matrix of item-scale and item-summary scale correlations were examined. We considered satisfactory if the item-own scale correlation and corrected item-total correlation was ≥ 0.30.

#### Reproducibility

The reproducibility on the LTQL questionnaire was assessed though a test-retest study. The questionnaire of LTQL were send again via mail ten days after to 43 patients to answer again. In order to measure the agreement between the two wets of responses, the intraclass correlation coefficients (ICC) was calculated using a two-way mixed effects model. Values higher than 0.7 are considered acceptable (31).

### Validity

#### Structural validity

To assess the structural validity of the questionnaire, different approaches were used. Firstly, we conducted EFA with promax rotation and CFA for categorical data to confirm the original structure put forth by the developers (16). This analysis aimed to determine if the 34 items in the questionnaire aligned with the proposed four-factor structure (domains), namely somatic concerns, philosophical/spiritual view of life, fitness and social support. Further, DIMTEST procedure was used to test the number of underlying factors in the questionnaire (32), and based on the results, the EFA and CFA were reconsidered. Secondly, we employed Rasch analysis to evaluate the unidimensionality and item functioning of each specific domain in the questionnaire. This analysis helps determine if the items within each dimension measure a single underlying construct effectively.

In the EFA, an item was considered to be in the factor if the factor loading and communality were ≥ 0.40 (33). In the CFA, we employed the robust weighted least squares estimator to estimate the model parameters. We calculated several fit indices to evaluate the goodness of fit of the model (34–37). The first fit index is the root mean square error of approximation (RMSEA), which provides a measure of how well the model fits the observed data. A RMSEA value < 0.08 is considered acceptable model fit and values < 0.06 are considered good model fit. The second set of fit indices includes the Tucker-Lewis Index (TLI) and Comparative Fit Index (CFI). Both indices assess the relative fit of the model by comparing it to a baseline model. Values > 0.95 indicate good model fit and values >0.90 indicate acceptable model fit. Further, we also examined the normed chi-square, considering values < 2 acceptable. However, because the chi-square test is sensitive to sample size, it was not used as a primary indicator of model fit. The standardized root mean square residual (SRMR) was also examined, for which a value < 0.08 was considered good fit. Additionally, we examined the factor loadings, which indicate the strength and direction of the relationships between the observed variables (items) and the underlying factors. Factor loadings ≥ 0.40 are generally considered acceptable, indicating that the items adequately reflect the factors they are intended to measure. Lagrange multiplier test, which identifies paths or covariances that should possibly be added to the model to improve the fit, was used when the model needed modification (36).

In the Rasch analysis, we utilized the polytomous Partial Credit Model due to the ordinal nature of the response scales in the questionnaire (38, 39), and it does not assume that the transition from category to category in the item is constant, allowing different probability of positive response from one category to another (40–42). We conducted separate analyses for each domain of the questionnaire to ensure that each domain was measuring a single underlying construct, which is a fundamental requirement in Rasch models (43). To assess unidimensionality, we employed two fit indices: the mean square information-weighted statistic (infit) and the outlier-sensitive statistic (outfit). Infit and outfit values between 0.6 and 1.4 are indicative of a good fit between the observed data and the model (44). We also conducted a principal component analysis (PCA) of the residuals to further evaluate unidimensionality. If the first domain was the only one with eigenvalues > 2, it would indicate that unidimensionality was not violated (45). To determine the position of items along the measured dimension, we examined the item separation index. This index provides an indication of the ability of the LTQL to discriminate between different levels of the measured construct. A value >2.0 is considered acceptable and comparable to a reliability of 0.80, suggesting that the items are effectively measuring distinct levels of the construct (43).

In order to assess local dependency, we examined the residual correlations between items within a domain of the questionnaire. The effect size of the model fit, MADaQ3, was provided as a summary measure of all pairs of Yen’s Q3 residual correlations. Values closed to 0 support the assumption of local independence (46). Identifying local dependency helps ensure that the items are measuring distinct aspects of the construct and are not overly redundant. We also examined the functioning of the rating scale categories for each item. It is important to have a clear and progressive level of difficulty across the response categories, indicating that higher response options correspond to higher levels of the underlying construct (43). If the response categories were found to be disordered, meaning that higher response options did not consistently reflect increases in the construct. The item-person map for each domain was also provided, in which both individuals and items are presented in the same logit scale. To detect differential item functioning (DIF), which occurs when different groups within the sample respond differently to individual items (38), we compared different levels of the trait bases on age group (< 65 vs. ≥ 65 years). Age can influence how a person faces and adapts their experiences and emotional aspects regarding BC survivorship. We used the Mantel-Haenszel test considering a statistically significant at P<0.05 to indicate noticeable DIF (47) and the following cut-off points: if |DIF| ≥ 0.43 logits, it is considered slightly to moderate DIF; and if |DIF| ≥ 0.64 logits, we considered moderate to large DIF (48).

In addition, we have explored the careless responding by means of the standardized log-likelihood (l_z_), which is a person-fit statistic based on item response theory that quantifies the discrepancy between the expected and empirical likelihood for each individual (49–51), and it is useful to detect unexpected responses. The l_z_ statistic asymptotically follows a standard normal distribution, and we used the theoretical cutoff of -2.326 (one-tailed significance test with α = 0.01). That is, a response pattern would be flagged if l_z_ is less than -2.326.

Finally, a CFA was performed considering the items that fitted the Rasch model, in order to compare the results of the structural validity once the misfitting items had been excluded.

#### Convergent validity

To assess the convergent validity of the LTQL questionnaire, we used Spearman’s Correlation coefficient to examine the relationships between LTQL domains and other validated questionnaires, such as QLACS (18), EORTC-QLQ-BR23 (19), HADS (20). We hypothesized that LTQL total score should have negative high correlations with other total scores of other QoL measures such as QLACS and EORTC-QLQ-BR23 and with other emotional measures such as HADS anxiety and depression. On the other hand, we hypothesized that somatic concerns domain of the LTQL should have a negative high correlation with certain domains of QLACS (fatigue, cognitive problems, negative feelings, sexual problems, pain, appearance concerns, distress recurrence) and domains of HADS (depression and anxiety). We have been unable to study convergent validity for the other LTQL domains (philosophical/spiritual view of life, fitness, and social support) due to the lack of adequate dimensions or scales in our study. We considered convergent validity as moderate when 0.3<r<0.49 and high if r≥0.50 (52).

#### Known-groups validity

Known-groups validity of the LTQL was examined by comparing the LTQL total score and domain scores among groups based on the type of treatment (yes/no lumpectomy, yes/no mastectomy, etc.). For this analysis, we used t-test or non-parametric Wilcoxon tests. We hypothesized that patients who had more aggressive treatment would have lower LTQL. Furthermore, to assess the magnitude of group differences, the effect size was calculated as the mean difference divided by the pooled standard deviation. Cohen’s benchmarks were used to classify the magnitude of effect sizes: <0.20 being considered not significant; 0.20 to 0.49 small, 0.50 to 0.79 moderate, and ≥0.80 large (53).

All effects were considered statistically significant at P<0.05. The statistical analyses were performed with IBM SPSS Statistics for Macintosh (Version 25.0 macOS 10.12.x (Sierra); IBM), SAS for Windows (Version 9.4; SAS Institute Inc., Cary, NC, USA, 2016), Mplus (Version 6.1; Muthén & Muthén, 1998-2010), Winsteps (Version 3.71.0.1; John M. Linacre, 2011), and RStudio (Version 1.4.1106; © 2009-2021 RStudio, PBC). The R package EFA.dimensions version 0.1.8.1 (Brian P. O’Connor, 2023) was used for the DIMETEST procedure and the R package PerFit version 1.4.6 (Jorge N. Tendeiro, 2021) for the l_z_ person-fit statistic to explore careless responding.

## Results

During the translation-back-translation process, the researchers encountered two points where they sought advice from the original authors. The first point involved difficulties in capturing the conceptual meaning of certain expressions, which were resolved through consultation with the original authors and incorporated into the final translated version. The second point of consultation related to item 16, which contained the concept of “subtle cues”. The researchers identified a problem with the meaning of this concept in the Spanish context. Through consensus, the item was modified to enhance clarity, improve understanding, and ensure accuracy within the Spanish version. Additional details on the content validation of the LTQL can be found in the referenced publication (54).

During the recruitment period, a total of 340 women were included in the field study. The main characteristics of the sample are summarized in **Table 1**. Mean scores with SDs for each domain and the total score of the LTQL are presented in **Table 2**. None of the domains showed floor or ceiling effects, indicating that less than 15% of patients scored at the minimum or maximum score, respectively, for any given domain.

**Table 1:** Demographic and disease characteristics of the study subjects (N=340).

Variable	N (%)
Gender (female)	340 (100%)
Age mean (SD) range	58.3 (10.4) 31–99
Age group <65 years old ≥65 years old	248 (72.9) 92 (27.1)
Marital status Married/couple Single Widowed Divorced/separated	205 (60.3%) 34 (10%) 35 (10.3%) 66 (19.4%)
Education < Primary school Primary school Secondary school University	20 (5.9%) 81 (23.8%) 126 (37.1%) 113 (33.2%)
Employment status Never worked Retired Unemployed Employed	17 (5.0%) 145 (42.6%) 28 (8.2%) 150 (44.1%)
Years of survival mean (SD) range	9.2 (3.9) 5–24
Type of treatment Lumpectomy Mastectomy Sentinel node extraction Emptying the armpit Reconstructive surgery Radiation therapy (without surgery) Chemotherapy Radiation therapy after surgery Hormone therapy or hormone therapy Target, directed, or molecular therapy External breast prosthesis	209 (61.5%) 148 (43.5%) 269 (79.1%) 137 (40.3%) 128 (37.6%) 34 (10%) 276 (81.2%) 283 (83.2%) 261 (76.8%) 14 (4.1%) 61 (17.9%)

SD, standard deviation.

**Table 2:** Descriptive data and reliability analysis for LTQL generic summary scale and domains.

Domains	N	Mean (SD)	Score range*	Floor effect (%)	Ceiling effect (%)	Cronbach’s alpha	McDonald’s omega	ICC (95% CI) (n=43)
Domain 1. Somatic concerns	340	2.87 (0.87)	0–4	0.3	2.9	0.89	0.89	0.96 (0.93 to 0.98)
Domain 2. Philosophical/Spiritual View of Life	340	2.57 (0.87)	0.27–4	0.3	3.5	0.86	0.85	0.72 (0.54 to 0.84)
Domain 3. Fitness	340	2.21 (1.21)	0–4	6.5	8.8	0.89	0.89	0.82 (0.68 to 0.90)
Domain 4. Social Support	340	2.53 (1.04)	0–4	2.4	8.8	0.83	0.84	0.77 (0.61 to 0.87)
LTQL generic summary	340	2.64 (0.58)	0.68–4	0.3	0.3	0.86		0.81 (0.67 to 0.90)

ICC, intraclass correlation coefficients; CI, confidence intervals; SD, standard deviation.

*Possible score rang: 0-4 (4: higher quality of life)

### Reliability

#### Internal consistency

The Cronbach’s alpha and McDonald’s omega coefficients are shown in **Table 2** and they were above 0.7 in all domains and the summary scale, supporting the internal consistency. As can be seen in **Table 3**, in LTQL domains, all corrected item-domain and item-summary scale correlations were above 0.30, with similar values in item-domain correlation (range: 0.38 to 0.82) and in the item-summary scale correlations (range: 0.36 to 0.82). Missing data was 0% in all items.

**Table 3:** Results of the Exploratory Factor Analysis and item-domain and item-summary scale correlations correcting for overlap for each LTQL domains.

Items	EFA^*^					Item-domain correlation	Item-summary scale correlation
	Factor loadings				Communality		
	F1	F2	F3	F4			
Somatic concerns							
05	**0.57**	0.0001	0.24	0.21	0.53	0.51	0.53
07	**0.59**	-0.01	-0.004	-0.03	0.35	0.48	0.52
08	**0.72**	-0.03	-0.08	0.06	0.53	0.65	0.65
11	**0.56**	-0.04	0.09	-0.08	0.32	0.46	0.49
12	**0.67**	-0.04	0.05	0.02	0.47	0.56	0.61
14	**0.74**	0.1	-0.01	-0.16	0.53	0.62	0.64
19	**0.71**	-0.01	-0.05	0.05	0.51	0.65	0.63
22	**0.63**	-0.06	-0.15	-0.07	0.41	0.55	0.55
23	**0.76**	0.01	-0.02	0.11	0.60	0.69	0.70
24	**0.61**	-0.05	0.17	-0.03	0.42	0.50	0.55
25	**0.70**	0.04	0.06	-0.12	0.49	0.58	0.62
28	**0.73**	0.02	-0.02	-0.16	0.53	0.64	0.65
32	**0.56**	-0.03	-0.07	0.01	0.31	0.47	0.49
34	**0.47**	0.05	**-0.55**	0.11	0.43	0.38	0.36
Philosophical/Spiritual View of Life							
02	-0.02	**0.52**	-0.14	0.17	0.31	0.43	0.42
03	0.17	**0.48**	0.17	0.23	0.50	0.55	0.57
09	0.04	**0.70**	-0.09	-0.12	0.42	0.50	0.48
10	-0.01	**0.54**	-0.02	0.22	0.41	0.53	0.55
13	0.07	**0.56**	0.03	0.22	0.46	0.59	0.58
16	0.10	**0.61**	0.08	0.21	0.55	0.65	0.65
18	-0.05	**0.74**	-0.01	-0.13	0.51	0.55	0.56
20	-0.08	**0.73**	0.11	-0.22	0.56	0.59	0.57
26	-0.01	**0.66**	0.02	0.18	0.55	0.68	0.68
27	-0.10	**0.72**	0.02	-0.15	0.51	0.57	0.55
30	-0.10	**0.50**	0.01	-0.01	0.25	0.45	0.41
Fitness							
04	0.06	-0.02	**0.80**	0.06	0.68	0.71	0.71
15	-0.09	0.05	**0.81**	-0.08	0.65	0.68	0.69
17	0.04	0.12	**0.70**	-0.02	0.56	0.64	0.65
21	0.10	-0.002	**0.82**	0.03	0.72	0.76	0.76
29	0.01	-0.09	**0.91**	0.05	0.80	0.82	0.82
Social Support							
01	-0.05	-0.03	0.02	**0.88**	0.76	0.73	0.75
06	0.004	0.08	-0.01	**0.88**	0.77	0.73	0.77
31	-0.16	0.30	-0.02	**0.41**	0.33	0.40	0.42
33	-0.09	-0.03	-0.01	**0.88**	0.74	0.71	0.73

^*^The percentage of variance explained was 51.40%. The correlation between the factors ranged from -0.06 to 0.38.

In bold values of factor loadings > 0.40.

#### Reproducibility

Considering test-retest reliability, range from 0.72 to 0.96 (**Table 2**) in the domains scales.

### Validity

#### Structural validity

**Figure 1:**
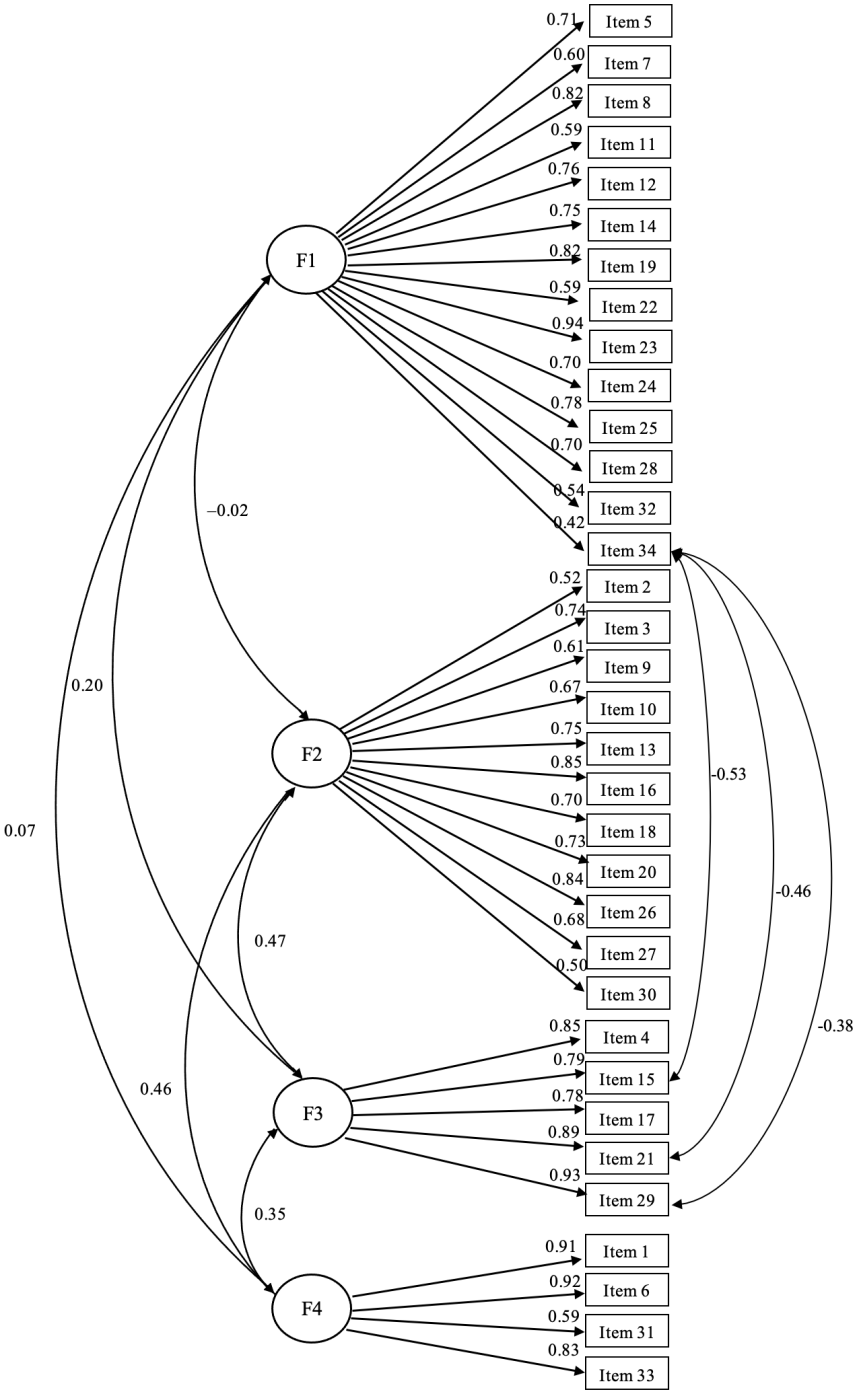
Confirmatory factor analysis for categorical data of the LTQL questionnaire. Domain somatic concerns (F1), domain philosophical/spiritual view of life (F2), domain fitness (F3), domain social support (F4). Standardized parameters are shown. Covariance was specified between the errors of the following three pair of items: items 34 and 15, items 34 and 29, and items 34 and 21. Fit indexes are as follows: χ2 = 1561.11, degrees of freedom = 518; RMSEA (90% CI) = 0.077 (0.073 – 0.081); CFI=0.909; TLI=0.901; SRMR=0.102.

The EFA provided factor loadings ranging from 0.41 to 0.91, exceeding the benchmark of 0.40 (**Table 3**). The item 34 showed high factor loadings in both “Somatic concern” and “Fitness” domains. The communality values were also higher than 0.40 for most items, except for items 7, 11, 32, 2, and 31, which were higher than 0.30, and item 30 with communality of 0.25. The percentage of variance explained by the four factors was 51.40%. The results of the CFA for the four factors (domains) model provided acceptable fit indices (**Figure 1**). Based on the Lagrange multiplier test, covariances between the errors of the following three pair of items were considered to improve the model fit: items 34 and 15, items 34 and 29, and items 34 and 21. We have already seen in EFA results that item 34 had a very high factor loading in the Fitness domain to which items 15, 29 and 21 belong. For this CFA (**Figure 1**) the RMSEA value was 0.077, less than 0.80, and both TLI and CFI, 0.901 and 0.909 respectively, were higher than the threshold of 0.90, just above the threshold to consider an acceptable model fit. The normed chi-square was 3.01, exceeding the threshold of 2, and the SRMR was 0.102, exceeding the threshold of 0.08. Further, all domain loading was above 0.40, ranging from 0.42 to 0.94, and were statistically significant (*P*<0.0001).

**Figure 2:**
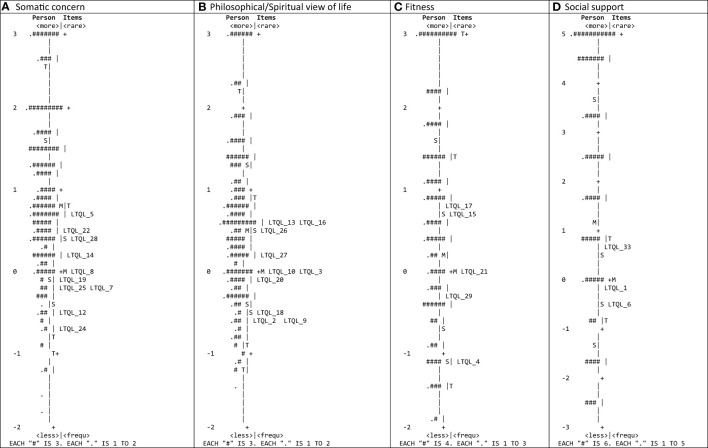
Item-person map of each LTQL domain: **(A)** somatic concerns, **(B)** philosophical/spiritual view of life, **(C)** fitness, and **(D)** social support. Both individuals and items are presented in the same logit scale.

The DIMTEST procedure suggested five factors, instead of four. Therefore, the EFA was explored considering five factors (**Supplementary Table S1**). Results were similar with the main difference that the “Philosophical/Spiritual view of life” domain is divided into two factors: items 3, 10, 13, 16 and 26, on the one hand (domain 2A with items related to post-cancer growth), and items 2, 9, 18, 20, 27 and 30, on the other hand (domain 2B with items related to spiritual guidance). Besides, the item 34 also showed high factor loadings in both “Somatic concern” and “Fitness” domains, and the communality values were lower than 0.40 for items 7, 11, 32 and 31, although higher than 0.30, except for item 30 with communality of 0.26. The percentage of variance explained by the five factors increased to 56.42%. The results of the CFA for the five factors model provided slightly better fit indices (**Supplementary Table S1**). The RMSEA value was 0.068, the TLI and CFI were 0.924 and 0.930, respectively, the normed chi-square was 2.55, and the SRMR was 0.089. Further, all domain loading was above 0.40, ranging from 0.42 to 0.93, and were statistically significant (*P*<0.0001).The results of the Rasch analyses for each domain are shown in **Table 4**. Regarding the Somatic Concerns domain, four items showed misfit (items 11, 23, 32 and 34) and were thus removed from the scale. In the Philosophical/Spiritual View of Life domain only one item showed misfit and was removed (item 30). The same applies to the Social Support domain, with one item removed (item 31) due to misfit. After removing those items, the rest of the items conforming each domain supported the unidimensionality with infit and outfit statistics between 0.6 and 1.4. The PCA of the residuals did not yield additional factors with eigenvalues higher than 2 in any of the four domains, and therefore the unidimensionality was also supported. The item separation indexes were high for all domains, ranging from 3.94 for the Social Support domain to 9.56 for the Fitness domain, indicating reliability higher than 0.80. The functioning of the rating scale categories was also adequate in all item of all domains, except in item 20, in which the first two categories were found disordered, although with a minimal difference (**Supplementary Table S2**). The item and person location for each domain are shown in **Figure 2**. Regarding local independence, MADaQ3 effect sizes ranged from 0.091 in Fitness domain to 0.227 in Philosophical/Spiritual view of life domain. Besides, regarding DIF between age groups analysis, although we found two items from the Philosophical/Spiritual View of Life domain (items 3 and 20) with significant DIF, the magnitude of the difference was lower than the threshold of 0.43, and therefore, we did not considered relevant (**Supplementary Table S3**).

**Table 4:** Severity levels, standard errors, and goodness-of-fit indices of each LTQL domain using Rasch analysis.

Items	Item description	δ (logit)	SE	Infit MNSQ	Outfit MNSQ
Domain 1. Somatic concerns					
5	I am satisfied with my body as it is now	0.65	0.05	1.06	1.13
7	I feel more susceptible to other illnesses since having had cancer	–0.20	0.06	1.21	1.36
8	I am self-conscious about my body since my cancer	0.00	0.06	1.04	0.91
11	I have to raise my arm or foot on a pillow so my rings or shoes fit all day since my cancer treatment	Removed			
12	I have difficulty finding suitable clothing since my cancer	–0.51	0.07	1.09	0.92
14	I continue to have pain since my cancer treatment	0.17	0.05	0.86	0.90
19	I feel dissatisfied with the way I look since my cancer	–0.06	0.06	1.01	0.93
22	My eyesight has gotten worse since my cancer treatment	0.48	0.05	1.08	1.12
23	I have difficulty accepting my body since my cancer	Removed			
24	My social life is less satisfying since having cancer	–0.71	0.07	1.08	0.95
25	In the past week, I have experienced pain related to having had cancer	–0.22	0.06	0.94	0.86
28	I have numbness and/or tingling since my cancer treatment	0.40	0.05	1.00	0.96
32	I frequently feel distressed with pain/discomfort because it reminds me of my cancer	Removed			
34	I have had to adjust the way I exercise since my cancer	Removed			
Domain 2. Philosophical/Spiritual View of Life					
2	I have a better idea about what serious illness is since having had cancer	–0.64	0.06	1.12	1.10
3	I feel a guiding energy in my life which has my best interest in mind	–0.03	0.05	0.99	0.94
9	Since having had cancer, I have a greater appreciation for the time I spend with my friends and family	–0.56	0.06	1.38	1.32
10	I follow my inner voice when making health decisions	0.01	0.05	1.09	1.10
13	I have intuitive experiences that reassure me about my health care choices	0.59	0.05	0.85	0.86
16	I receive subtle cues that give me confidence in my health decisions	0.60	0.05	0.70	0.73
18	I am sympathetic with family/friends who have major illnesses, such as heart or kidney disease since my cancer	–0.55	0.06	1.05	1.05
20	Since having had cancer, I tend to notice things in nature more, such as sunsets, raindrops and spring flowers	–0.13	0.05	1.22	1.21
26	I feel an inner direction that helps me make wise decisions	0.55	0.05	0.74	0.70
27	I have become closer with some family members/friends since having had cancer	0.18	0.05	1.24	1.27
30	Since having had cancer, I don’t take life’s little things for granted	Removed			
Domain 3. Fitness					
4	Exercise helps me feel healthy	–1.14	0.08	1.05	0.89
15	I exercise more frequently	0.71	0.07	1.13	1.04
17	Regular exercise keeps me healthy, so I am less likely to get cancer again	0.75	0.07	1.29	1.23
21	Exercise helps decrease my fatigue	–0.02	0.07	0.94	0.93
29	Exercise helps me feel energetic	–0.30	0.07	0.71	0.69
Domain 4. Social Support					
1	I think I could be helpful to others who have recently been diagnosed with cancer	–0.15	0.10	0.82	0.83
6	I would like to be a resource person to others who have recently been diagnosed with cancer	–0.44	0.10	0.98	0.95
31	I would find it beneficial to speak with other long-term cancer survivors	Removed			
33	I think that I have support and understanding to offer other long-term cancer survivors	0.59	0.10	1.17	1.15

δ, level of severity, with higher values indicating higher severity; SE, standard error; MNSQ, mean square fit statistic.

Item separation index of each model: 6.77 for the Somatic Concerns domain, 7.70 for the Philosophical/Spiritual View of Life domain, 9.56 for the Fitness domain, and 3.94 for the Social Support domain.

MADaQ3 effect size of each domain: 0.113 for the Somatic Concerns domain, 0.227 for the Philosophical/Spiritual View of Life domain, 0.091 for the Fitness domain, and 0.116 for the Social Support domain.

We also performed the Rasch analysis for the two subscales of the “Philosophical/Spiritual view of life” suggested by the five-factor EFA solution (**Supplementary Table S4**). The results were similar to those found for the “Philosophical/Spiritual view of life” total domain, except that the functioning of the rating scale categories of item 20 was adequate and the item 20 did not present DIF by age group. However, we found significant DIF in items 3 and 26, with values slightly higher than the threshold of 0.43 to consider not relevant, although lower than 0.64 (**Supplementary Table S4**). Regarding local independence, MADaQ3 effect sizes were much lower, 0.074 and 0.054, respectively.

Regarding the careless responding examination for each domain, the respondents with critical l_z_ values were minimal, ranging from 1.18% of the participants (4 patients) in the Fitness domain to 3.82% (13 patients) in the Philosophical/Spiritual view of life domain, with l_z_ values very closed to the threshold of -2.326. Further, when we analyzed the careless responding in the two subscales of the Philosophical/Spiritual view of life domain proposed by EFA, the careless respondents were even lower (0.58% and 0.88%, respectively). Therefore, we concluded that careless responding was not relevant.

The results of the CFA for the four factors after excluding the six misfitting items, provided acceptable fit indices. The RMSEA value was 0.080 (90% CI, 0.075 – 0.086), and both TLI and CFI, 0.915 and 0.924 respectively, were higher than the threshold of 0.90, just above the threshold to consider an acceptable model fit. The normed chi-square was 3.19, exceeding the threshold of 2, and the SRMR was 0.097. Further, all domain loadings were above 0.40, ranging from 0.52 to 0.93, and were statistically significant (*P*<0.0001).

The results of the CFA for the five-factor structure, after excluding the six misfitting items, shown slightly better results. The RMSEA value was 0.066 (90% CI, 0.061 – 0.072), and both TLI and CFI, 0.95 and 0.94 respectively, almost reach the threshold of 0.95 to be considered satisfactory fit. The normed chi-square was 2.49, exceeding the threshold of 2, the SRMR was 0.080, and factor loadings ranged from 0.59 to 0.93.

#### Convergent validity

**Table 5** shows results on the convergent validity analysis. As we hypothesized, LTQL total score was negatively correlated with the total score of other QoL scales, such as QLACS (r=-0.39) and EORTC-QLQ-BR23 (r=-0.50), and with other emotional measures such as HADS anxiety (r=-0.45) and HADS depression (r=-0.57).

**Table 5:** Correlation between LTQL generic summary and different measures.

	Correlation *r*	Convergent measures
LTQL generic summary	-0.39 -0.50 -0.57 -0.45	QLACS generic summary EORTC-QLQ-BR23 generic summary HADS depression HADS anxiety
LTQL domain 1: Somatic concerns	-0.54 -0.55 -0.50 0.33 -0.54 -0.74 -0.68 -0.61 -0.70 -0.51 -0.50	QLACS Fatigue QLACS Cognitive problems QLACS Negative feelings QLACS Positive feelings QLACS Sexual problems QLACS Pain QLACS Appearance concern QLACS Distress recurrence EORT-QLQ-BR23 HADS Depression HADS anxiety

All correlation coefficient were significantly different from 0 (p<0.05).

QLACS, Quality of Life in Adults Cancer Survivors; EORTC-QLQ-BR23, European Organization for Research and Treatment of Cancer Quality of Life questionnaire module for Breast Cancer Patients; HADS, Hospital Anxiety and Depression Scale.

Regarding the somatic concerns domain of the LTQL was negatively correlated with certain domains of QLACS, such as fatigue (r=-0.54), cognitive problems (r=-0.55), negative feelings (r=-0.50), sexual problems (r=-0.54), pain (r=-0.74), appearance concerns (r=-0.68), distress recurrence (r=-0.61), domains of HADS, such as depression (r=-0.51) and anxiety (r=-0.50), and EORT-QOL-BR23 (r=-0.70).

#### Known-groups validity

Known-groups validity was evaluated across all domains, revealing statistically significant differences solely within domain 1 (somatic concerns). Patients who underwent with Lumpectomy demonstrated higher scores in somatic concerns (t=-2.02, p=0.044) and did those who did not undergo mastectomy (t=2.314, p=0.021). Conversely, no statistical significant differences (p>0.05) were observed between domains of LTQL and various other treatment types, including sentinel node extraction, axillary lymph node dissection, plastic reconstructive surgery, absence of surgical radiotherapy, chemotherapy before or after surgery, hormonal therapy, hormonal therapy before and/or after surgery, targeted therapy, or molecular therapy, or the use of external breast prostheses.

## Discussion

This study focused on the validation of the Spanish version of the LTQL questionnaire using both classical psychometric methods and Rasch approach. While the LTQL questionnaire was originally developed for United States context, the findings of this study demonstrated that it is a valid and reliable instrument for assessing QoL in long-term BC female survivors in the Spanish population. By examining the reliability and validity of the LTQL questionnaire in another language, this study aimed to contribute valuable information for its application in diverse linguistic and cultural settings. The results suggest that the Spanish version of the LTQL questionnaire can be confidently used in research and clinical practice to assess QoL in Long-term BC female survivors.

The internal consistency of the Spanish version of the LTQL questionnaire demonstrated good reliability, with Cronbach’s alpha coefficients ranging from 0.83 to 0.89 for all domains and summary scale, and McDonald’s omega coefficients ranging from 0.84 to 0.89. These values are similar to those reported in the original instrument. Furthermore, the item-domain and item-summary scale correlations also showed satisfactory values, exceeding the accepted threshold of 0.30 (16, 17). The test-retest reliability, assessed in stable patients, was found to be appropriate in all domains, further supporting the instrument’s reliability (31).

Ceiling and floor effects were not observed in any of the summary domains, indicating that the questionnaire adequately captured the full range of responses. Additionally, the absence of missing data in all items further strengthens the reliability of the instrument.

Convergent validity was found to be good in the LTQL, as evidenced by the significant correlations between the LTQL scores and other validated questionnaires.

The EFA provided satisfactory results regarding factor loadings, except for item 34 which shown high loading in two domains, “Somatic concern” and “Fitness”. Items 7, 11, 32, 2, and 31 showed lower communalities, between 0.30 and 0.40, but the item of most concern was item 30 with a communality of 0.25, indicating that it was not well represented by the factors. The findings of the CFA, although did not meet the threshold to consider a satisfactory model fit, they exceeded the thresholds for acceptable model fit, supporting the structural validity of the questionnaire, and confirming the existence of four subscales, which aligns with the results reported by the original authors (16, 17). Both RMSEA, and CFI and TLI indexes were acceptable, and also the factor loadings. The normed chi-square was not satisfactory, but we did not consider a primary indicator because it is influenced by the sample size (55). However, DIMTEST procedure suggested a five-factor structure (Philosophical/Spiritual view of life domain was divided into two factors: domain 2A with items related to post-cancer growth and domain 2B with items related to spiritual guidance), and the results of the CFA for five dimensions were somewhat better, with lower RMSEA and SRMR, and higher CFI and TLI. Rasch analysis identified six misfitting items in the LTQL questionnaire, including items from the Somatic Concerns domain (items 11, 23, 32 and 34), Philosophical/Spiritual View of Life domain (item 30), and Social Support domain (item 31). However, the results of the CFAs after removing the six misfitting items, for both four and five factor structures, showed that the exclusion of these six items did not seem to improve the model fit. Further, due to the lack of comparable studies, it is difficult to make direct comparisons with previous Rasch analysis of the LTQL. Consequently, we believe that further studies would be necessary to make the decision of eliminate items from the Spanish version of the model.

Regarding known-groups validity, with patients treated with Lumpectomy and those who did not undergo mastectomy reporting significantly higher QOL in domain 1 (somatic concerns).

This study acknowledges several limitations that should be taken into consideration. Firstly, the findings may not be generalizable to individuals with other types of cancer, as the study focused specifically on BC patients. Secondly, the study primarily included long-term female survivors, which may restrict the generalizability of the findings to short-term survivors or to male survivors. Thirdly, the EFA and CFA to examine the structural validity were performed using the same sample, which presents unfortunate consequences, such us artificial overfitting (56). Lastly, the assessment of convergent validity was limited by the availability of appropriate dimensions in the other instruments used for comparison. The lack of relevant dimensions in the existing instruments may have hindered a comprehensive evaluation of convergent validity across all domains of the LTQL questionnaire. Future studies should aim to include instruments that encompass a wider range of dimensions to obtain a more comprehensive assessment of convergent validity. Moreover, besides having to be valid and reliable, an instrument must also be responsive to changes to be useful. The responsiveness of the LTQL has not yet been explored.

Considering these limitations, it is important to interpret the results of this study with caution and to consider the specific context and characteristics of the studied population when applying the LTQL questionnaire in other settings or with different cancer populations. Further research with diverse samples and cancer types is needed to validate the generalizability of the findings and to enhance the understanding of QoL in cancer survivors.

## Conclusions

This study provides evidence supporting the acceptable reliability and validity of the Spanish version of the LTQL questionnaire, indicating its suitability for assessing the QoL in long-term BC female survivors. While the questionnaire proves valuable for measuring QoL in this specific population, we advocate for further investigation before considering the elimination of items from the Spanish version of the model.

The Spanish version of the LTQL questionnaire can confidently be employed to evaluate the QoL of long-term BC survivors in the Spanish-speaking population, providing a comprehensive assessment across multiple relevant domains.

Its utilization enables healthcare professionals and researchers to gain insights into QoL outcomes, identify areas of concern, tailor interventions, and monitor changes in QoL over time, thereby contributing to a more comprehensive understanding of the impact of BC on survivors’ lives and facilitating the development of targeted support strategies.

We recommend that healthcare providers and researchers consider integrating the Spanish version of the LTQL questionnaire into their practice and studies to assess QoL in long-term BC survivors and enhance their overall well-being and survivorship experiences.

Additionally, further research with diverse samples and cancer types is needed to validate the generalizability of these findings and deepen our understanding of QoL in cancer survivors.

## Data availability statement

The raw data supporting the conclusions of this article will be made available by the authors, without undue reservation.

## Ethics statement

The studies involving humans were approved by Institutional review board of the Nuestra Señora de Candelaria University Hospital (approval number CHUNSC-2019-04). The studies were conducted in accordance with the local legislation and institutional requirements. The participants provided their written informed consent to participate in this study.

## Author contributions

BLS: Conceptualization, Data curation, Formal analysis, Funding acquisition, Investigation, Methodology, Project administration, Resources, Software, Supervision, Validation, Visualization, Writing – original draft, Writing – review & editing. ABG: Data curation, Formal analysis, Software, Validation, Writing – review & editing. APM: Writing – review & editing. ME: Writing – review & editing. ATC: Writing – review & editing. CFS: Writing – review & editing. UBS: Writing – review & editing. PPP: Writing – review & editing. SGS: Writing – review & editing. RVL: Writing – review & editing. MTM: Writing – review & editing.

## In Memoriam

This article is dedicated to Dr. Antonio Escobar Martínez, who left us on October 14, 2020. An inveterate researcher in Health Services at the Kronikgune Institute created in the Basque Country and whose research activity has been linked to the Research Network on Health Services in Chronic Diseases-REDISSEC (Spain) in recent years. Thank you very much for the teachings. Rest in peace.
