# Supplementary material for: Cross-cultural adaptation, reliability and validity of the Spanish version of the long-term quality of life questionnaire
Source: Front Oncol. 2024 Mar 19;14:1375125. doi: 10.3389/fonc.2024.1375125 (PMC10985178; doi:10.3389/fonc.2024.1375125)
Supplement: Supplementary file 1 [file Table_1.docx]

**Supplementary Table S1.** Results of the Exploratory Factor Analysis and Confirmatory Factor Analysis of the LTQL questionnaire considering five factors.

| Items | EFA^*^ | | | | | | | | | CFA^**^ | | | | | | |
| --- | --- | --- | --- | --- | --- | --- | --- | --- | --- | --- | --- | --- | --- | --- | --- | --- |
|  | Factor loadings | | | | | | Communality | | Factor loadings | | | | | | |  |
|  | F1 | F2 | F3 | F4 | F5 |  | | F1 | | | F2 | F3 | F4 | F5 |  |  |
| Somatic concerns |  |  |  |  |  |  | |  | | |  |  |  |  |  |  |
| 05 | **0.58** | 0.03 | -0.02 | 0.25 | 0.24 | 0.54 | | **0.71** | | |  |  |  |  |  |  |
| 07 | **0.58** | -0.07 | 0.09 | -0.01 | -0.05 | 0.35 | | **0.60** | | |  |  |  |  |  |  |
| 08 | **0.73** | 0.03 | -0.07 | -0.07 | 0.09 | 0.54 | | **0.82** | | |  |  |  |  |  |  |
| 11 | **0.54** | -0.11 | 0.10 | 0.08 | -0.11 | 0.33 | | **0.59** | | |  |  |  |  |  |  |
| 12 | **0.68** | 0.05 | -0.11 | 0.06 | 0.06 | 0.48 | | **0.76** | | |  |  |  |  |  |  |
| 14 | **0.73** | 0.01 | 0.13 | -0.02 | -0.18 | 0.54 | | **0.75** | | |  |  |  |  |  |  |
| 19 | **0.72** | 0.06 | -0.09 | -0.04 | 0.09 | 0.53 | | **0.82** | | |  |  |  |  |  |  |
| 22 | **0.63** | -0.01 | -0.07 | -0.15 | -0.05 | 0.42 | | **0.59** | | |  |  |  |  |  |  |
| 23 | **0.77** | 0.09 | -0.08 | -0.01 | 0.15 | 0.63 | | **0.94** | | |  |  |  |  |  |  |
| 24 | **0.61** | -0.03 | -0.04 | 0.17 | -0.01 | 0.42 | | **0.70** | | |  |  |  |  |  |  |
| 25 | **0.70** | 0.07 | -0.02 | 0.07 | -0.10 | 0.49 | | **0.78** | | |  |  |  |  |  |  |
| 28 | **0.72** | -0.06 | 0.10 | -0.03 | -0.18 | 0.53 | | **0.71** | | |  |  |  |  |  |  |
| 32 | **0.55** | -0.06 | 0.04 | -0.07 | -0.01 | 0.31 | | **0.54** | | |  |  |  |  |  |  |
| 34 | **0.45** | -0.04 | 0.13 | **-0.56** | 0.07 | 0.44 | | **0.42** | | |  |  |  |  |  |  |
| Philosophical/Spiritual View of Life |  |  |  |  |  |  | |  | | |  |  |  |  |  |  |
| 02 | 0.03 | **0.60** | -0.05 | -0.12 | 0.27 | 0.44 | |  | | | **0.61** |  |  |  |  |  |
| 09 | 0.09 | **0.80** | -0.05 | -0.06 | 0.002 | 0.58 | |  | | | **0.71** |  |  |  |  |  |
| 18 | -0.004 | **0.79** | 0.01 | 0.02 | -0.02 | 0.63 | |  | | | **0.80** |  |  |  |  |  |
| 20 | -0.04 | **0.73** | 0.07 | 0.13 | -0.13 | 0.63 | |  | | | **0.84** |  |  |  |  |  |
| 27 | -0.06 | **0.71** | 0.10 | 0.03 | -0.07 | 0.57 | |  | | | **0.80** |  |  |  |  |  |
| 30 | 0.004 | **0.42** | 0.17 | 0.02 | 0.01 | 0.26 | |  | | | **0.58** |  |  |  |  |  |
| 03 | 0.12 | 0.01 | **0.67** | 0.13 | 0.09 | 0.61 | |  | | |  | **0.80** |  |  |  |  |
| 10 | -0.06 | 0.05 | **0.72** | -0.05 | 0.07 | 0.55 | |  | | |  | **0.71** |  |  |  |  |
| 13 | 0.003 | -0.02 | **0.83** | -0.02 | 0.02 | 0.68 | |  | | |  | **0.79** |  |  |  |  |
| 16 | 0.04 | 0.05 | **0.81** | 0.04 | 0.04 | 0.73 | |  | | |  | **0.88** |  |  |  |  |
| 26 | -0.08 | 0.07 | **0.85** | -0.02 | -0.001 | 0.76 | |  | | |  | **0.87** |  |  |  |  |
| Fitness |  |  |  |  |  |  | |  | | |  |  |  |  |  |  |
| 04 | 0.06 | -0.07 | 0.07 | **0.79** | 0.04 | 0.68 | |  | | |  |  | **0.85** |  |  |  |
| 15 | -0.08 | 0.09 | -0.04 | **0.81** | -0.05 | 0.66 | |  | | |  |  | **0.79** |  |  |  |
| 17 | 0.03 | -0.01 | 0.19 | **0.68** | -0.06 | 0.57 | |  | | |  |  | **0.78** |  |  |  |
| 21 | 0.11 | 0.01 | -0.01 | **0.82** | 0.04 | 0.73 | |  | | |  |  | **0.89** |  |  |  |
| 29 | 0.02 | -0.05 | -0.06 | **0.90** | 0.07 | 0.81 | |  | | |  |  | **0.93** |  |  |  |
| Social Support |  |  |  |  |  |  | |  | | |  |  |  |  |  |  |
| 01 | -0.02 | -0.03 | 0.02 | 0.03 | **0.88** | 0.79 | |  | | |  |  |  | **0.91** |  |  |
| 06 | 0.03 | -0.005 | 0.05 | -0.003 | **0.88** | 0.80 | |  | | |  |  |  | **0.92** |  |  |
| 31 | -0.13 | 0.29 | 0.06 | -0.01 | **0.44** | 0.37 | |  | | |  |  |  | **0.59** |  |  |
| 33 | -0.07 | -0.07 | 0.07 | -0.002 | **0.86** | 0.76 | |  | | |  |  |  | **0.83** |  |  |

^*^ The percentage of variance explained was 56.42%. The correlation between the factors ranged from -0.13 to 0.38.

^**^ Standardized parameters are shown. Covariance was specified between the errors of the following three pair of items: items 34 and 15, items 34 and 29, and items 34 and 21. Fit indexes are as follows: χ2 = 1311.36, degrees of freedom = 514; RMSEA (90% CI) = 0.068 (0.063 – 0.072); CFI=0.930; TLI=0.924; SRMR=0.089. The correlation between the factors ranged from -0.13 to 0.57.

**Supplementary Table S2.** Difficulty level of each category in each LTQL item by Rasch analysis.

| Items | Response categories | | | | |
| --- | --- | --- | --- | --- | --- |
|  | 0 | 1 | 2 | 3 | 4 |
|  | δ (SE) | δ (SE) | δ (SE) | δ (SE) | δ (SE) |
| Domain 1. Somatic concerns | | | | | |
| 5 | -0.19 (0.13) | 0.37 (0.08) | 0.75 (0.07) | 1.19 (0.08) | 1.99 (0.14) |
| 7 | -0.49 (0.19) | 0.12 (0.21) | 0.33 (0.11) | 0.66 (0.06) | 1.43 (0.08) |
| 8 | -0.45 (0.15) | 0.18 (0.08) | 0.28 (0.10) | 0.63 (0.06) | 1.57 (0.07) |
| 12 | -0.93 (0.23) | -0.02 (0.14) | 0.12 (0.08) | 0.54 (0.10) | 1.34 (0.07) |
| 14 | -0.40 (0.19) | -0.03 (0.09) | 0.44 (0.07) | 0.86 (0.07) | 1.72 (0.09) |
| 19 | -0.58 (0.21) | 0.10 (0.07) | 0.23 (0.08) | 0.66 (0.05) | 1.57 (0.08) |
| 22 | -0.23 (0.14) | 0.35 (0.08) | 0.59 (0.08) | 1.07 (0.09) | 1.83 (0.11) |
| 24 | -1.10 (0.35) | -0.27 (0.16) | 0.11 (0.10) | 0.37 (0.10) | 1.29 (0.07) |
| 25 | -0.64 (0.18) | -0.09 (0.13) | 0.26 (0.10) | 0.61 (0.08) | 1.49 (0.07) |
| 28 | -0.21 (0.11) | 0.17 (0.10) | 0.59 (0.09) | 0.95 (0.08) | 1.77 (0.09) |
| Domain 2. Philosophical/Spiritual View of Life | | | | | |
| 2 | -0.84 (0.43) | -0.46 (0.12) | -0.02 (0.08) | 0.41 (0.07) | 1.09 (0.10) |
| 3 | -0.67 (0.12) | -0.10 (0.07) | 0.24 (0.08) | 0.58 (0.07) | 1.43 (0.12) |
| 9 | -0.46 (0.16) | -0.38 (0.18) | -0.09 (0.10) | 0.25 (0.07) | 1.10 (0.08) |
| 10 | -0.55 (0.13) | -0.09 (0.09) | 0.25 (0.06) | 0.66 (0.06) | 1.37 (0.12) |
| 13 | -0.32 (0.09) | 0.02 (0.07) | 0.48 (0.06) | 0.92 (0.08) | 2.23 (0.20) |
| 16 | -0.47 (0.08) | 0.11 (0.07) | 0.49 (0.06) | 1.02 (0.07) | 2.35 (0.23) |
| 18 | -0.74 (0.18) | -0.34 (0.14) | -0.07 (0.11) | 0.29 (0.06) | 1.17 (0.09) |
| 20 | -0.36 (0.09) | -0.43 (0.13) | 0.02 (0.09) | 0.44 (0.09) | 1.31 (0.09) |
| 26 | -0.36 (0.08) | -0.04 (0.07) | 0.47 (0.05) | 0.89 (0.07) | 2.09 (0.16) |
| 27 | -0.24 (0.09) | -0.08 (0.15) | 0.19 (0.08) | 0.40 (0.06) | 1.57 (0.11) |
| Domain 3. Fitness | | | | | |
| 4 | -3.53 (0.20) | -1.75 (0.18) | -0.54 (0.10) | 0.17 (0.10) | 1.70 (0.12) |
| 15 | -1.58 (0.17) | -0.46 (0.10) | 0.43 (0.08) | 1.50 (0.11) | 2.85 (0.18) |
| 17 | -1.56 (0.17) | -0.27 (0.13) | 0.50 (0.11) | 1.21 (0.11) | 3 (0.17) |
| 21 | -2.31 (0.19) | -0.67 (0.11) | 0.03 (0.09) | 0.86 (0.09) | 2.34 (0.14) |
| 29 | -2.60 (0.17) | -0.90 (0.12) | -0.10 (0.08) | 0.65 (0.08) | 2.18 (0.12) |
| Domain 4. Social Support | | | | | |
| 1 | -5.02 (0.40) | -2.10 (0.20) | 0.31 (0.14) | 2.37 (0.15) | 5.03 (0.11) |
| 6 | -4.99 (0.31) | -1.87 (0.18) | 0.11 (0.15) | 2.09 (0.16) | 4.77 (0.13) |
| 33 | -3.60 (0.46) | -1.51 (0.20) | 0.77 (0.18) | 2.75 (0.14) | 5.45 (0.10) |

δ = level of difficulty; SE = standard error.

**Supplementary Table S3.** Severity levels of each LTQL item in each domain using Rasch analysis by age group and the Differential Item Functioning analysis.

| Items | DIF by age group | | | |
| --- | --- | --- | --- | --- |
|  | <65 years | ≥65 years |  |  |
|  | δ (SE) | δ (SE) | Diff. δ (SE) | Mantel-Haenszel *P* |
| Domain 1. Somatic concerns | | | | |
| 5 | 0.59 (0.06) | 0.83 (0.10) | 0.24 (0.12) | 0.4141 |
| 7 | -0.26 (0.07) | 0.05 (0.12) | 0.31 (0.14) | 0.2211 |
| 8 | 0.07 (0.06) | -0.29 (0.14) | -0.36 (0.15) | 0.1274 |
| 12 | -0.51 (0.07) | -0.48 (0.15) | 0.03 (0.17) | 0.4444 |
| 14 | 0.17 (0.06) | 0.23 (0.11) | 0.06 (0.13) | 0.7462 |
| 19 | -0.02 (0.06) | -0.25 (0.14) | -0.23 (0.15) | 0.4860 |
| 22 | 0.46 (0.06) | 0.55 (0.10) | 0.09 (0.12) | 0.8903 |
| 24 | -0.68 (0.08) | -0.85 (0.19) | -0.17 (0.20) | 0.8082 |
| 25 | -0.19 (0.07) | -0.35 (0.14) | -0.16 (0.16) | 0.5464 |
| 28 | 0.40 (0.06) | 0.33 (0.11) | -0.07 (0.13) | 0.6573 |
| Domain 2. Philosophical/Spiritual View of Life | | | | |
| 2 | -0.69 (0.08) | -0.56 (0.10) | 0.13 (0.13) | 0.2292 |
| 3 | 0.09 (0.06) | -0.29 (0.10) | -0.38 (0.12) | 0.0003 |
| 9 | -0.64 (0.08) | -0.40 (0.10) | 0.24 (0.13) | 0.1315 |
| 10 | 0.01 (0.07) | -0.02 (0.09) | -0.02 (0.11) | 0.8103 |
| 13 | 0.63 (0.06) | 0.48 (0.09) | -0.15 (0.11) | 0.2501 |
| 16 | 0.65 (0.06) | 0.47 (0.09) | -0.18 (0.11) | 0.1176 |
| 18 | -0.55 (0.08) | -0.52 (0.10) | 0.02 (0.13) | 0.9951 |
| 20 | -0.23 (0.07) | 0.05 (0.09) | 0.28 (0.11) | 0.0237 |
| 26 | 0.51 (0.06) | 0.64 (0.09) | 0.13 (0.11) | 0.2636 |
| 27 | 0.18 (0.06) | 0.18 (0.09) | 0 (0.11) | 0.7725 |
| Domain 3. Fitness | | | | |
| 4 | -1.14 (0.09) | -1.14 (0.14) | 0 (0.17) | 0.9543 |
| 15 | 0.63 (0.08) | 0.92 (0.13) | 0.29 (0.15) | 0.1142 |
| 17 | 0.79 (0.08) | 0.65 (0.13) | -0.15 (0.15) | 0.3692 |
| 21 | 0.05 (0.08) | -0.20 (0.13) | -0.25 (0.15) | 0.1997 |
| 29 | -0.33 (0.08) | -0.23 (0.13) | 0.10 (0.15) | 0.5651 |
| Domain 4. Social Support | | | | |
| 1 | -0.23 (0.12) | 0.04 (0.19) | 0.27 (0.23) | 0.2120 |
| 6 | -0.44 (0.12) | -0.44 (0.19) | 0 (0.23) | 0.9148 |
| 33 | 0.66 (0.12) | 0.40 (0.19) | -0.27 (0.22) | 0.3414 |

δ = level of severity; DIF = Differential Item Functioning; Diff. = Difference in difficulty level between <65 and ≥65 years.

**Supplementary Table S4.** Severity levels, standard errors, and goodness-of-fit indices of the two sub-scales of the Phylosophical/Spiritual view of life domain using Rasch analysis

| Items | Item description | δ  (logit) | SE | Infit MNSQ | Outfit MNSQ | DIF by age group | | | |
| --- | --- | --- | --- | --- | --- | --- | --- | --- | --- |
|  |  |  |  |  |  | <65 years | ≥65 years |  |  |
|  |  |  |  |  |  | δ (SE) | δ (SE) | Diff. δ (SE) | Mantel-Haenszel *P* |
| Domain 2A. Post-cancer growth | |  |  |  |  |  |  |  |  |
| 2 | I have a better idea about what serious illness is since having had cancer | –0.36 | 0.07 | 1.11 | 1.40 | -0.36 (0.08) | -0.38 (0.11) | -0.02 (0.14) | 0.9886 |
| 9 | Since having had cancer, I have a greater appreciation for the time I spend with my friends and family | –0.26 | 0.07 | 1.08 | 1.10 | -0.30 (0.08) | -0.20 (0.11) | 0.10 (0.14) | 0.6338 |
| 18 | I am sympathetic with family/friends who have major illnesses, such as heart or kidney disease since my cancer | –0.25 | 0.07 | 0.82 | 0.84 | -0.20 (0.08) | -0.35 (0.11) | -0.15 (0.14) | 0.1690 |
| 20 | Since having had cancer, I tend to notice things in nature more, such as sunsets, raindrops and spring flowers | 0.24 | 0.06 | 0.93 | 0.91 | 0.18 (0.07) | 0.37 (0.11) | 0.18 (0.13) | 0.2114 |
| 27 | I have become closer with some family members/friends since having had cancer | 0.63 | 0.06 | 0.98 | 0.92 | 0.67 (0.07) | 0.55 (0.11) | -0.11 (0.13) | 0.6359 |
| 30 | Since having had cancer, I don’t take life’s little things for granted | Removed |  |  |  |  |  |  |  |
| Domain 2B. Spiritual guidance | |  |  |  |  |  |  |  |  |
| 3 | I feel a guiding energy in my life which has my best interest in mind | -0.65 | 0.07 | 1.24 | 1.19 | -0.50 (0.09) | -1.03 (0.13) | -0.54 (0.16) | 0.0047 |
| 10 | I follow my inner voice when making health decisions | -0.58 | 0.07 | 1.36 | 1.32 | -0.62 (0.09) | -0.50 (0.13) | 0.12 (0.15) | 0.7579 |
| 13 | I have intuitive experiences that reassure me about my health care choices | 0.43 | 0.07 | 0.93 | 0.96 | 0.43 (0.08) | 0.43 (0.12) | 0 (0.15) | 0.9081 |
| 16 | I receive subtle cues that give me confidence in my health decisions | 0.45 | 0.07 | 0.73 | 0.72 | 0.47 (0.08) | 0.40 (0.12) | -0.07 (0.15) | 0.5293 |
| 26 | I feel an inner direction that helps me make wise decisions | 0.36 | 0.07 | 0.81 | 0.76 | 0.23 (0.08) | 0.68 (0.12) | 0.45 (0.15) | 0.0005 |

δ = level of severity, with higher values indicating higher severity; SE = standard error; MNSQ = mean square fit statistic.

Item separation index of each model: 5.75 for the domain 2A. Post-cancer growth, and 6.83 for the for the domain 2B. Spiritual guidance

MADaQ3 effect size of each domain: 0.074 for the domain 2A. Post-cancer growth, and 0.054 for the for the domain 2B. Spiritual guidance.
