# Supplementary material for: Cross-cultural adaptation, reliability and validity of the Spanish version of the long-term quality of life questionnaire
Source: Front Oncol. 2024 Mar 19;14:1375125. doi: 10.3389/fonc.2024.1375125 (PMC10985178; doi:10.3389/fonc.2024.1375125)
Supplement: Supplementary file 2 [file DataSheet_1.docx]

**SYNTAX**

**Cronbach Alpha Coefficient**

- Statistical program: IBM SPSS Statistics for Macintosh (Version 25.0 macOS 10.12.x (Sierra); IBM).
- Syntax example for the Domain 1:

RELIABILITY

**/**VARIABLES=LTQL_5

LTQL_7

LTQL_8

LTQL_11

LTQL_12

LTQL_14

LTQL_19

LTQL_22

LTQL_23

LTQL_24

LTQL_25

LTQL_28

LTQL_32

LTQL_34

/SCALE('ALL VARIABLES') ALL

/MODEL=ALPHA.

**McDonald’s omega coefficient**

- Statistical program: IBM SPSS Statistics for Macintosh (Version 25.0 macOS 10.12.x (Sierra); IBM).
- Syntax example for the Domain 1:

RELIABILITY

/VARIABLES=LTQL_5 LTQL_7 LTQL_8 LTQL_11 LTQL_12 LTQL_14 LTQL_19 LTQL_22 LTQL_23

LTQL_24 LTQL_25 LTQL_28 LTQL_32 LTQL_34

/SCALE('ALL VARIABLES') ALL

/MODEL=OMEGA

/STATISTICS=DESCRIPTIVE SCALE

/SUMMARY=TOTAL.

**Intraclass correlation coefficients**

- Statistical program: IBM SPSS Statistics for Macintosh (Version 25.0 macOS 10.12.x (Sierra); IBM).
- Syntax example for the Domain 1:

RELIABILITY

/VARIABLES=LTQL_Factor1_T1 LTQL_Factor1_T2

/SCALE('ALL VARIABLES') ALL

/MODEL=ALPHA

/ICC=MODEL(MIXED) TYPE(ABSOLUTE) CIN=95 TESTVAL=0.

**Correlation coefficients**

NONPAR CORR

/VARIABLES=LTQL_Factor_1 LTQL_Factor_2 LTQL_Factor_3 LTQL_Factor_4 LTQL_Total QLACS_Total

QLACS_Negative_feelings QLACS_Positive_feelings QLACS_Cognitive_problems QLACS_Sexual_problems

QLACS_Pain QLACS_Fatigue QLACS_Social_avoidance QLACS_Generic_domain QLACS_Appearance_concern

QLACS_Finalcial_problems QLACS_Distress_recurrence QLACS_Family_distress QLACS_Benefits

QLACS_Specific_domins HADS_ansiedad HADS_depresion HADS_total EORT_QLQBR23

/PRINT=SPEARMAN TWOTAIL NOSIG

/MISSING=PAIRWISE.

**Exploratory Factor Analysis**

- Statistical program: SAS for Windows (Version 9.4; SAS Institute Inc., Cary, NC, USA, 2016).
- Syntax example for the 4-factor solution:

PROC FACTOR DATA=DATA

SIMPLE

SCREE

METHOD=PRIN

PRIORS=ONE

NFACT=4

ROTATE=PROMAX(3);

VAR LTQL_1-LTQL_34;

RUN;

**Confirmatory Factor Analysis**

- Statistical program: Mplus (Version 6.1; Muthén & Muthén, 1998-2010).
- Syntax example for the 4-factor solution:

TITLE: LTQL: CFA categorical data

DATA: FILE IS C:\Users\Public\Documents\............\data.dat;

VARIABLE: NAMES ARE LTQL_1-LTQL_34;

CATEGORICAL LTQL_1-LTQL_34;

USEVARIABLES ARE LTQL_1-LTQL_34;

MODEL: f1 BY LTQL_5 LTQL_7 LTQL_8 LTQL_11 LTQL_12

LTQL_14 LTQL_19 LTQL_22 LTQL_23 LTQL_24

LTQL_25 LTQL_28 LTQL_32 LTQL_34;

f2 BY LTQL_2 LTQL_3 LTQL_9 LTQL_10 LTQL_13

LTQL_16 LTQL_18 LTQL_20 LTQL_26 LTQL_27

LTQL_30;

f3 BY LTQL_4 LTQL_15 LTQL_17 LTQL_21 LTQL_29;

f4 BY LTQL_1 LTQL_6 LTQL_31 LTQL_33;

LTQL_34 WITH LTQL_15;

LTQL_34 WITH LTQL_29;

LTQL_34 WITH LTQL_21;

ANALYSIS: ESTIMATOR = WLSMV;

MODEL = NOMEANSTRUCTURE;

OUTPUT: STAND RES MOD(ALL) TECH4;

**DIMTEST procedure**

- Statistical program: RStudio (Version 1.4.1106; © 2009-2021 RStudio, PBC), using the R package EFA.dimensions version 0.1.8.1 (Brian P. O'Connor, 2023).

install.packages("EFA.dimensions",dependencies=TRUE)

library(EFA.dimensions)

EFA.dimensions::DIMTESTS(datos, corkind='polychoric', display=2)

**Rasch analysis**

- Statistical program: Winsteps (Version 3.71.0.1; John M. Linacre, 2011).
- Syntax example for the Domain 1

&INST

TITLE = "RASCH LTQL: FACTOR 1"

;Input Data Format

NAME1 = 1 ; column of start of person information

NAMLEN = 5 ; maximum length of person information

ITEM1 = 6 ; column of first item-level response

NI = 34 ; number of items = test length

XWIDE = 1 ; number of columns per response

PERSON = Person ; Persons are called ...

ITEM = Item ; Items are called ...

DATA = 'C:\Users\Public\Documents\..........\raschdef.txt'

IDFILE=*

15-34

*

CODES = "01234

CLFILE = *

0 = 0

1 = 1

2 = 2

3 = 3

4 = 4

*

DISCRIM=YES

IFILE = 'C:\Users\Public\Documents\...........'

&END

LTQL_5

LTQL_7

LTQL_8

LTQL_11

LTQL_12

LTQL_14

LTQL_19

LTQL_22

LTQL_23

LTQL_24

LTQL_25

LTQL_28

LTQL_32

LTQL_34

LTQL_2

LTQL_3

LTQL_9

LTQL_10

LTQL_13

LTQL_16

LTQL_18

LTQL_20

LTQL_26

LTQL_27

LTQL_30

LTQL_4

LTQL_15

LTQL_17

LTQL_21

LTQL_29

LTQL_1

LTQL_6

LTQL_31

LTQL_33

END LABELs

**Standardized log-likelihood *l_z_* person-fit statistic**

- Statistical program: RStudio (Version 1.4.1106; © 2009-2021 RStudio, PBC), using the R package PerFit version 1.4.6 (Jorge N. Tendeiro, 2021).

install.packages("PerFit",dependencies=TRUE)

library(PerFit)

lzpoly.out <- lzpoly(data,Ncat=5)

lzpoly.out
